# Preserved vegetable consumption and its association with mortality among 440,415 people in the China Kadoorie Biobank

**DOI:** 10.1186/s12916-023-02829-3

**Published:** 2023-04-05

**Authors:** Pan Zhuang, Fei Wu, Xiaohui Liu, Fanghuan Zhu, Yin Li, Jingjing Jiao, Yu Zhang

**Affiliations:** 1 Department of Gastroenterology, The First Affiliated Hospital, Zhejiang University School of Medicine, 79 Qingchun Road, Hangzhou, 310003 Zhejiang China; 2 Zhejiang Key Laboratory for Agro-Food Processing, College of Biosystems Engineering and Food Science, Zhejiang University, 866 Yuhangtang Road, Hangzhou, 310058 Zhejiang China; 3 Department of Nutrition, School of Public Health, Zhejiang University School of Medicine, 866 Yuhangtang Road, Hangzhou, 310058 Zhejiang China; 4 Department of Endocrinology, The Second Affiliated Hospital, Zhejiang University School of Medicine, 88 Jiefang Road, Hangzhou, 310009 Zhejiang China

**Keywords:** Cardiovascular disease mortality, Hemorrhagic stroke mortality, Esophageal cancer mortality, Preserved vegetables, China Kadoorie Biobank

## Abstract

**Background:**

Fresh vegetable consumption has been associated with lower incidence of cardiovascular disease (CVD). However, whether preserved vegetable consumption is linked with CVD and mortality remains unclear. This study aimed to assess the associations of preserved vegetable consumption with all-cause and cause-specific mortality.

**Methods:**

A total of 440,415 participants free of major chronic diseases, aged 30–79 years, were enrolled from 10 diverse regions in China between 2004 and 2008 and were followed up for an average of 10 years. Preserved vegetable consumption was assessed using a validated food frequency questionnaire. Cause-specific hazard models with the consideration of competing risk from various deaths were performed to calculate hazard ratios (HRs) and 95% confidence intervals (CIs) of mortality.

**Results:**

During 4,415,784 person-years of follow-up, we documented 28,625 deaths. After adjustment for major risk factors, preserved vegetable consumption was marginally associated with higher CVD mortality (*P* = 0.041 for trend and *P* = 0.025 for non-linearity) but not associated with cancer mortality and total mortality. For specific causes of death, consuming preserved vegetables was associated with higher hemorrhagic stroke mortality. The multivariable-adjusted HRs (95% CIs) of hemorrhagic stroke mortality compared with non-consumers were 1.32 (1.17–1.50) for 1–3 days/week and 1.15 (1.00–1.31) for regular consumers (≥4 days/week) (*P* = 0.006 for trend and *P* < 0.001 for non-linearity). In addition, regular preserved vegetable consumption was associated with increased risk of digestive tract cancer mortality [HR (95% CI): 1.13 (1.00–1.28); *P* = 0.053 for trend] and esophageal cancer mortality [HR (95% CI): 1.45 (1.17–1.81); *P* = 0.002 for trend].

**Conclusions:**

Frequent consumption of preserved vegetables was associated with higher risk of mortality from hemorrhagic stroke and esophageal cancer in China. Our findings suggest limiting preserved vegetable consumption might be protective for premature death from hemorrhagic stroke and digestive tract cancer.

**Supplementary Information:**

The online version contains supplementary material available at 10.1186/s12916-023-02829-3.

## Background

Salting as a food processing method is commonly used to prolong the expiration date of perishable foods [1]. Cucumbers, red cabbages, beets, or carrots soaked with sugar and vinegar plus red pepper were common in Western countries. In China, approximately 0.68 billion people consume preserved vegetables [2], which are mainly made by putting fresh vegetables into brine and tons are exported overseas year after year [3]. Nonetheless, a recent meta-analysis of cohort studies showed that each 40 g/day increment in pickled vegetable intake was associated with a 15% higher risk of gastric cancer [4]. Evidence from a case–control study showed that frequent consumption of preserved vegetables was associated with higher risk of esophageal cancer and esophageal squamous cell carcinoma (ESCC) [5, 6]. However, limited data specifically focused on the associations with longevity despite a 10% higher all-cause mortality for salt-preserved vegetable consumption in a small-scale cohort study of old Chinese (aged ≥80 years) [7]. Furthermore, the associations of preserved vegetable consumption with cardiovascular disease (CVD) morbidity and mortality have not been assessed in China, where almost 94 million Chinese people suffer from CVD in 2016 and CVD mortality contributes to more than 40% of all deaths in 2013 [8, 9]. To fill these gaps, we examined a large prospective cohort of 440,415 participants to assess the association of preserved vegetable intake with all-cause, CVD, and cancer mortality in China.

## Methods

### Study population

The China Kadoorie Biobank study (CKB) is a prospective nationwide cohort study and jointly conducted by the University of Oxford and the Chinese Academy of Medical Sciences, which has been previously described in detail [10]. Briefly, the study was conducted in 10 geographically different regions, including five urban (Qingdao, Harbin, Haikou, Suzhou, and Liuzhou) and five rural (Sichuan, Gansu, Henan, Zhejiang, and Hunan) areas, in China to show the diversity in risk exposure and disease patterns considering population stability, quality of death and disease registries, local commitment, and capacity. Between June 2004 and July 2008, all residents aged 30–79 years from 100–150 administrative units in each area were invited to participate in the study and ultimately 512,900 individuals were recruited with written informed consent. Each qualified participant completed an interviewer-administered laptop-portable questionnaire consisting of 11 sections, including general demographic characteristics, diet, and behavioral lifestyle factors such as alcohol consumption, smoking and physical activity, domestic indoor air pollution, medical history and current medication, sleeping and mental status (using Composite International Diagnostic Interview Short-Form), reproductive history (for women), and physical examination [10]. After excluding the persons with wrong personal information (*n* = 176), those with missing BMI (*n* = 2), and individuals with chronic diseases (CVD, cancer, diabetes, and chronic obstructive pulmonary disease, *n* = 72,307), a total of 440,415 eligible participants (177,478 men and 262,937 women) were finally included in the current analysis. The data request from the current study was approved by the CKB Access Team.

### Dietary assessment and covariates

Dietary data covered 12 major food groups in China, including rice, wheat, other staple food (e.g., corn and millet), red meat, poultry, fish or seafood, fresh eggs, fresh vegetables, soybean products, preserved vegetables (e.g., pickled cabbage, pickled garlic, and pickled radish), fresh fruits, and dairy products (milk and yogurt), with five categories of consumption frequency (daily, 4–6 days/week, 1–3 days/week, monthly, or never/rarely). Participants with the frequency of ≥4 days/week habitual consumption were regarded as regular consumers in the current analysis. To check the reproducibility of dietary survey responses, the questionnaire interview was conducted again within a year after the baseline survey in a subsample of 926 participants [11]. The Spearman coefficient of reproducibility for preserved vegetable consumption was 0.54 [12].

The other personal information was also self-reported and collected at baseline, including demographic characteristics (age, race/ethnicity, educational levels, household annual income, occupation, and marital status), lifestyle factors (smoking status, physical activity, alcohol intake, and tea consumption), personal and family medical history (self-rated health status, CVD, cancer, diabetes, stroke, and hypertension), and drug use (anti-hypertensive medication and lipid-lowering medication). Weight and height were measured during physical examination using anthropometric methods. Body mass index (BMI) was defined as body weight (kg) divided by height (m) squared. Metabolic equivalent task hours per week (MET-h/wk) of physical activity for each participant was calculated by the Compendium of Physical Activities [13]. Well-trained clinicians performed spot random blood glucose testing using the SureStep Plus System (Johnson & Johnson). Blood pressure was measured at least twice with the use of an automated digital blood pressure monitor (model UA-779, A&D Medical) after at least a 5-min rest in a seated position; the mean of two satisfactory measurements was used for analyses [14]. A healthy diet score was calculated to assess individual long-term diet given fresh vegetable intake at least each day (median), fruit intake at least 1–3 days each week (median), soybean intake at least 1–3 days each week (median), wholegrain intake (other staples such as corn and millet) at least 4 days each week (median), egg intake at least 1–3 days each week (median), fish intake at least 4–6 days each week (median), and red meat intake at most 1–3 days each week (median). We granted 1 point for each favorable diet component, and the total diet score ranged from 0 to 7 [15, 16]. A diet score of 4 or more was regarded as a healthy diet [17].

### Ascertainment of mortality

Information on all-cause and cause-specific mortality of all the participants was obtained periodically by the disease surveillance points system of Chinese Center for Disease Control and Prevention (CDC), checked with the national health insurance system electronically linked with all hospitalizations, and confirmed with street committees or village administrators [18]. Causes of death were coded according to the International Classification of Diseases, 10^th^ Revision (ICD-10) by well-trained staffs who were unknown of the baseline characteristics of the study participants (Additional file 1: Table S1). The three main outcome measures that were examined were cardiovascular death (ICD-10 codes I00 to I25, I28 to I88, and I95 to I99), cancer death (codes C00 to C97), and overall death. Follow-up duration was calculated from the date of entry to death, the date of lost to follow-up, or December 31, 2016, whichever occurred earlier. By January 1, 2017, only 3898 (0.8%) individuals were lost to follow-up and censored in analyses.

### Statistical analyses

Baseline characteristics of the enrolled participants were described as means and standard deviations or percentages according to each category of preserved vegetable consumption. We used multiple linear regression models to estimate the marginal mean values and 95% confidence intervals (CIs) for BMI, waist circumference, blood pressure, and blood glucose according to the frequency of preserved vegetable consumption, adjusted for age (10 categories), sex, study area (10 regions), marital status (never married, married, divorced/separated, or widowed), BMI (in kg/m^2^; continuous; for non-BMI outcomes), household annual income (in yuan/year; <10,000, 10,000–19,999, 20,000–34,999, or ≥35,000), survey season, highest education level (no formal school, primary school, middle or high school, or college and above), physical activity (MET-h/wk), smoking status (non-smoker, occasional smoker, former smoker, or current smoker), alcohol drinking (non-drinker, occasional drinker, former drinker, or current drinker), family history of cancer (yes or no) and family history of CVD (yes or no), and consumption of red meat, poultry, eggs, fish, soybeans, fruit, dairy (never/rarely, monthly, 1–3 days/week, or regularly), and fresh vegetables (daily or less than daily). Cause-specific hazard models [19] which take competing risks into consideration were used to calculate hazard ratios (HRs) and 95% CIs for mortality across categories of preserved vegetable consumption and were sequentially adjusted for the same covariates as those reported above except that the analysis was stratified by age-at-risk (5-year intervals), sex, and study area (10 regions). The proportional hazards assumption was checked by the Kolmogorov-type supremum test. The linear trend was tested by fitting the ordinal preserved vegetable variables as continuous in the models. For significant relationships indicated by P trend, we further tested the non-linearity using a likelihood ratio test which compared the model with only the linear term of preserved vegetable consumption with the model with both the linear and the cubic spline terms. Given that the proportion of excess CVD and hemorrhagic stroke mortality associated with preserved vegetable consumption might be attributed to the elevation of blood pressure induced by sodium, we further conducted the mediation analysis [20] to estimate the mediation proportion with 95% CI of high blood pressure for preserved vegetable consumption.

We also performed several sensitivity analyses by excluding deaths occurred within initial 2 years of follow-up to minimize reverse causality bias. We also excluded participants with extreme BMI (<18.5 or >40 kg/m^2^) to see whether the findings were affected by extreme values. Other confounding factors were further considered, including further adjusting for the dose of cigarettes for current smokers [<20, 20-24, or ≥25 cigarettes (or equivalent) per day] and dose of alcohol for current drinkers (<140, 140 to <420, or ≥420 g of alcohol per week) [21, 22], history of hypertension, the use of vitamins, anti-hypertensive medication, and lipid-lowing medication, and self-rated poor health to test whether the results were stable. We further adjusted for a healthy diet score to assess whether the documented associations were due to the overall diet quality. We also evaluated whether the associations varied in subgroups stratified by age (<60 and ≥60 years), sex (men and women), BMI (<24 and ≥24 kg/m^2^), annual household income (below and above median), study area (urban and rural), physical activity (below and above median), smoking status (non/former smoker and current smoker), alcohol drinking (non-drinker and drinker), and blood pressure (hypertensive and non-hypertensive). *P* value for interaction was computed by the likelihood-ratio test.

Statistical analyses were performed with SAS 9.4 (SAS Institute, Cary, NC, USA). Tests were two-sided and the statistical significance was defined as *P* < 0.05.

## Results

### Preserved vegetable consumption and baseline characteristics

**Fig. 1:**
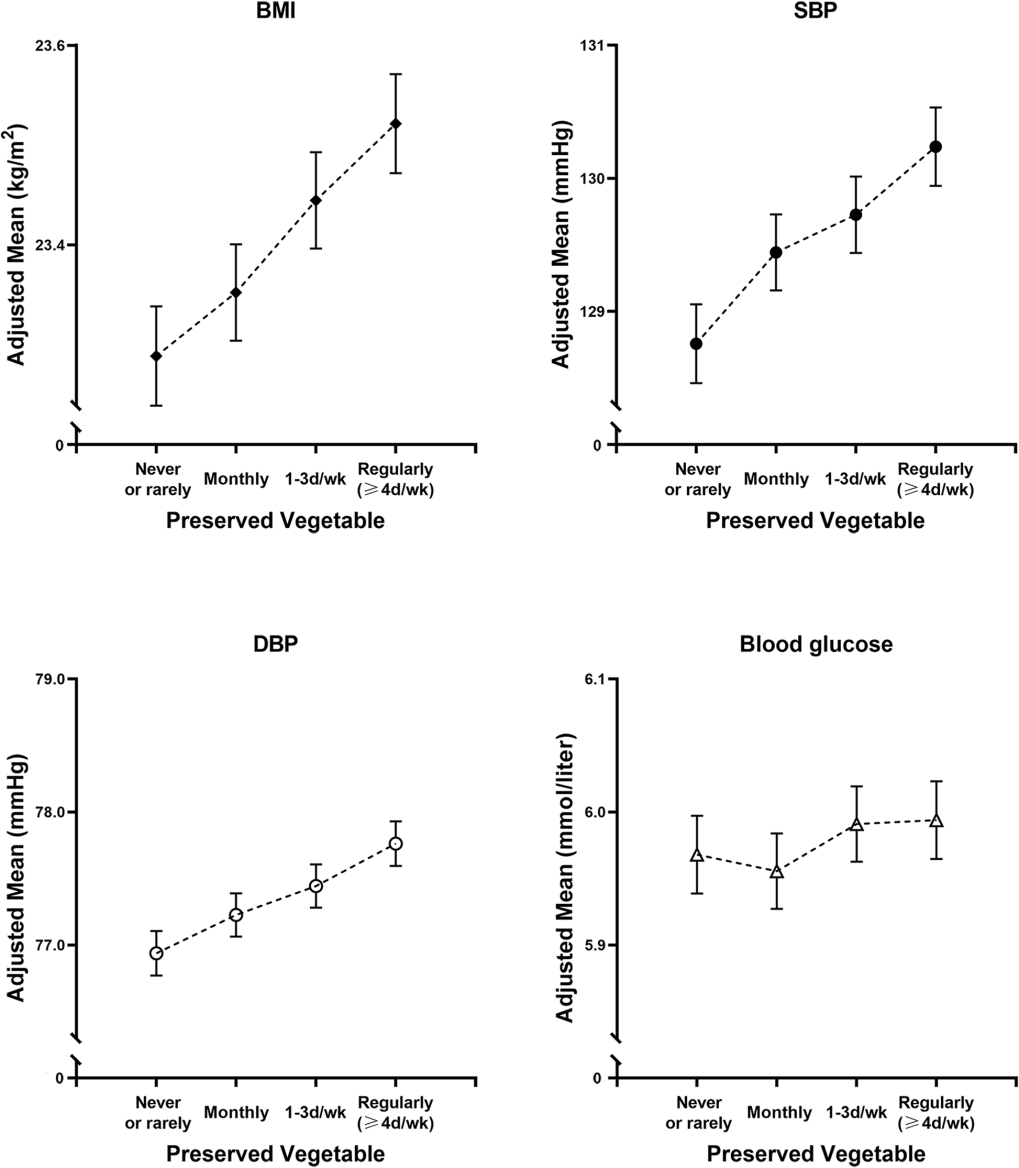
Adjusted mean BMI, blood pressure, and blood glucose according to frequency of preserved vegetable consumption. Adjusted means of BMI, systolic blood pressure, diastolic blood pressure, and blood glucose were estimated using linear regression models adjusted for age, sex, study area (10 regions), marital status, BMI (for non-BMI outcomes; continuous), household income, survey season, highest education level, physical activity, smoking, alcohol drinking, family history of cancer (yes or no), family history of CVD (yes or no), intake of red meat, poultry, eggs, fish, soybeans, fruit, dairy (never/rarely, monthly, 1–3 days/week, regularly), and fresh vegetables (daily or less than daily) at baseline. Vertical lines indicate 95% CIs. BMI, body mass index; CVD, cardiovascular disease; DBP, diastolic blood pressure; SBP, systolic blood pressure

During 4,415,784 person-years of follow-up (a mean of 10.0 years of follow-up), 28,625 deaths occurred, including 10,924 deaths from CVD, 10,392 from cancer, and 7,309 from other causes. Baseline characteristics of participants across categories of preserved vegetable consumption are shown in Table 1. At baseline, 22% of participants reported consumption of preserved vegetables ≥4 days/week (regular consumers) and 18% reported never/rarely consuming preserved vegetables (non-consumers), whereas 95% of participants daily consumed fresh vegetables. Compared with non-consumers, participants with a higher frequency of preserved vegetable consumption were wealthier and more likely to smoke (in men), while they were less likely to be educated. Moreover, they tended to consume more red meat and soybeans. Our participants had an overall mean BMI of 23.6 kg/m^2^, overall mean systolic blood pressure (SBP) of 130.1 mmHg, overall mean diastolic blood pressure (DBP) of 77.6 mmHg, and overall mean blood glucose level of 5.9 mmol/L. After controlling for multiple confounders, preserved vegetable consumption was positively correlated with BMI and blood pressure. Compared with participants who never or rarely consumed preserved vegetables, the BMI, SBP, and DBP of regular consumers were 0.2 kg/m^2^ and 1.5 and 0.8 mmHg higher, respectively, while there was no statistically significant difference between the adjusted means for blood glucose for regular consumers vs. non/rare consumers (Fig. 1).

**Table 1:** Baseline characteristics of participants by preserved vegetable consumption

Characteristics	Total	Frequency of preserved vegetable consumption			
		Never/rarely	Monthly	1–3 days/week	Regularly (≥4 days/week)
*n*	440,415	77,718	138,884	126,225	97,588
Age (year)	50.8 ± 10.3^a^	51.5 ± 10.8	50.8 ± 10.3	50.2 ± 10.2	51.1 ± 10.1
Male (%)	40.3	40.1	40.3	40.7	40.0
Body mass index (kg/m^2^)	23.6 ± 3.3	23.9 ± 3.4	23.4 ± 3.3	23.4 ± 3.3	24.1 ± 3.3
Married (%)	91.4	91.6	91.8	92.0	91.3
Urban residence (%)	43.2	49.9	34.0	34.8	61.7
Southern residence (%)^b^	59.8	46.2	66.6	63.4	56.4
≥High school (%)	21.4	28.9	20.7	19.3	19.3
Household income (≥35,000 yuan/year, %)	18.3	15.8	18.4	18.1	20.3
Physical activity (MET-h/wk)	153.6 ± 97.4	128.2 ± 91.7	149.7 ± 98.4	165.8 ± 95.7	163.5 ± 98.2
Smoking (%)					
Ex-regular (men)	11.4	12.2	11.7	10.5	11.6
Ex-regular (women)	0.6	0.7	0.4	0.6	1.0
Current regular (men)	62.7	55.9	61.1	64.7	67.8
Current regular (women)	2.0	2.0	1.3	1.8	3.3
Alcohol drinking (%)					
Ex-regular (men)	2.8	2.9	2.9	2.9	2.6
Ex-regular (women)	0.4	0.4	0.3	0.4	0.4
Current regular (men)	45.5	44.4	40.6	42.8	57.0
Current regular (women)	4.0	4.4	3.1	3.5	5.4
Family history of cardiovascular disease (%)	19.9	23.2	19.2	17.3	21.7
Family history of cancer (%)	16.3	18.9	15.2	13.4	19.7
Hypertension (%)	31.4	30.1	31.7	30.7	33.0
Self-rated poor health (%)	8.1	10.2	6.8	6.7	9.8
Systolic blood pressure (mmHg)	130.1 ± 20.8	129.1 ± 20.8	130.1 ± 20.9	129.9 ± 20.8	131.1 ± 20.7
Diastolic blood pressure (mmHg)	77.6 ± 11.1	76.9 ± 11.1	77.3 ± 11.1	77.6 ± 11.1	78.7 ± 11.0
Blood glucose (mmol/L)^c^	5.9 ± 1.9	5.9 ± 1.9	5.8 ± 1.8	5.8 ± 1.8	6.0 ± 2.0
Regular consumption of food (%)^d^					
Red meat	47.7	46.6	47.4	47.1	49.6
Poultry	1.4	1.3	1.2	1.5	1.5
Fish	9.2	11.5	10.3	6.6	9.0
Eggs	24.0	27.3	20.2	23.6	27.4
Dairy	10.9	15.4	8.2	9.5	12.8
Fresh vegetables	94.6	96.1	95.4	92.4	95.2
Fruit	28.2	30.4	22.0	27.7	36.1
Soybeans	9.6	8.3	8.6	10.8	10.4

*MET-h/wk* Metabolic equivalent task hours per week

^a^Data are percentages or mean ± standard deviation unless indicated otherwise

^b^Southern regions include Haikou, Suzhou, Liuzhou, Sichuan, Zhejiang, and Hunan

^c^7011 participants missed data of blood glucose

^d^At least 4 days/week consumption, except for fresh vegetables (daily)

### All-cause, CVD, and cancer mortality

Preserved vegetable consumption was not significantly associated with all-cause mortality after adjusting for age-at-risk (5-year intervals), sex, and region (*P* = 0.441 for trend) and further adjusting for demographic, lifestyle factors (model 2; *P* = 0.690 for trend), and dietary factors (model 3; *P* = 0.168 for trend). After multivariable adjustment, the level of preserved vegetable consumption was marginally and positively associated with CVD mortality (model 3; *P* = 0.041 for trend) whereas the relationship was not linear (*P* = 0.025 for non-linearity) (Table 2). Compared with non-consumption, the multivariable-adjusted HRs were 1.08 (95% CI: 1.01–1.15) for monthly intake, 1.12 (1.05–1.20) for 1–3 days per week intake, and 1.07 (0.99–1.15) for ≥4 days per week intake, respectively. For subtypes of CVD, preserved vegetable consumption was positively associated with hemorrhagic stroke mortality (*P* = 0.006 for trend and *P* < 0.001 for non-linearity). Compared with non-consumers, the adjusted HRs were 1.09 (0.97–1.23) for monthly consumers, 1.32 (1.17–1.50) for 1–3 days/week consumers, and 1.15 (1.00–1.31) for regular consumers. No associations of preserved vegetable intake were found for deaths from ischemic stroke and other CVD events (Table 3). The mediation analysis showed that 21.6% (12.5–34.7%) and 9.2% (6.1–13.7%) of increased CVD and hemorrhagic stroke mortality related to preserved vegetables was attributed to high blood pressure, respectively.

**Table 2:** Number of deaths and adjusted HRs (95% CIs) of all-cause mortality, CVD mortality, and cancer mortality according to preserved vegetable consumption

	Preserved vegetable consumption					
	Never/rarely	Monthly	1–3 days/week	Regularly (≥4 days/week)	*P* trend	*P* non-linearity
Person-years	771,737	1,392,939	1,260,552	990,556		
All-cause mortality						
Cases/*n*	5449/77,718	8909/138,884	8180/126,225	6087/97,588		
Model 1^a^	1	0.98 (0.94–1.02)	1.00 (0.96–1.04)	1.01 (0.97–1.06)	0.441	
Model 2^b^	1	1.00 (0.96–1.04)	1.02 (0.98–1.06)	1.00 (0.96–1.05)	0.690	
Model 3^c^	1	1.03 (0.99–1.07)	1.06 (1.02–1.11)	1.02 (0.98–1.07)	0.168	
CVD mortality						
Cases/*n*	2147/77,718	3454/138,884	3196/126,225	2127/97,588		
Model 1^a^	1	1.02 (0.96–1.08)	1.06 (0.99–1.13)	1.06 (0.99–1.14)	0.069	
Model 2^b^	1	1.04 (0.98–1.11)	1.08 (1.01–1.15)	1.05 (0.98–1.14)	0.102	
Model 3^c^	1	1.08 (1.01–1.15)	1.12 (1.05–1.20)	1.07 (0.99–1.15)	0.041	0.025
Cancer mortality						
Cases/*n*	1971/77,718	3034/138,884	2837/126,225	2550/97,588		
Model 1^a^	1	0.95 (0.89–1.01)	1.00 (0.94–1.07)	1.02 (0.95–1.09)	0.285	
Model 2^b^	1	0.96 (0.90–1.02)	1.01 (0.94–1.08)	1.00 (0.94–1.07)	0.587	
Model 3^c^	1	0.99 (0.93–1.05)	1.04 (0.97–1.11)	1.02 (0.95–1.10)	0.277	

*CI* Confidence interval, *CVD* Cardiovascular disease, *HR* Hazard ratio

^a^Model 1 was stratified by age-at-risk (5-year intervals), sex, and study area (10 regions)

^b^Model 2 was further adjusted for marital status (never married, married, divorced/separated, or widowed), body mass index (in kg/m^2^; continuous), household income (in yuan/year; <10,000, 10,000–19,999, 20,000–34,999, or ≥35,000), survey season, highest education level (no formal school, primary school, middle or high school, or college and above), physical activity (MET-h/wk), smoking (non-smoker, occasional smoker, ever-smoker, or current smoker), alcohol drinking (non-drinker, occasional drinker, ever-drinker, or current drinker), family history of cancer (yes or no), and family history of CVD (yes or no)

^c^Model 3 was further adjusted for intakes of red meat, poultry, eggs, fish, soybeans, fruit, dairy (never/rarely, monthly, 1–3 days/week, or regularly), and fresh vegetables (daily or less than daily) at baseline

**Table 3:** Number of deaths and adjusted HRs (95% CIs) of cause-specific mortality according to preserved vegetable consumption

Causes of death	Never/rarely		Monthly		1–3 days/week		Regularly (≥4 days/week)		*P* for trend	*P* nonlinearity
	No. of deaths	HR (95% CI)^a^	No. of deaths	HR (95% CI)^a^	No. of deaths	HR (95% CI)^a^	No. of deaths	HR (95% CI)^a^		
Ischemic heart disease	845	1	1271	1.06 (0.96–1.18)	1056	1.01 (0.90–1.13)	776	1.08 (0.96–1.22)	0.402	
Ischemic stroke	271	1	392	1.04 (0.86–1.26)	298	1.01 (0.82–1.25)	235	1.02 (0.81–1.28)	0.939	
Hemorrhagic stroke	522	1	1039	1.09 (0.97–1.23)	1291	1.32 (1.17–1.50)	690	1.15 (1.00–1.31)	0.006	<0.001
Other CVDs	509	1	752	1.11 (0.97–1.26)	551	1.08 (0.93–1.26)	426	0.99 (0.84–1.17)	0.992	
Stomach cancer	251	1	331	0.99 (0.83–1.19)	384	1.05 (0.87–1.27)	401	1.04 (0.86–1.26)	0.567	
Esophageal cancer	291	1	309	0.96 (0.81–1.14)	162	0.97 (0.77–1.23)	255	1.45 (1.17–1.81)	0.002	0.009
Colorectal cancer	115	1	241	1.24 (0.97–1.58)	228	1.18 (0.92–1.51)	175	1.00 (0.77–1.31)	0.758	
Digestive tract cancer	657	1	881	1.03 (0.92–1.15)	774	1.06 (0.94–1.20)	831	1.13 (1.00–1.28)	0.053	0.879
Non-digestive tract cancer	1314	1	2153	0.96 (0.89–1.04)	2063	1.03 (0.95–1.11)	1719	0.97 (0.89–1.06)	0.998	
Lung cancer	449	1	792	0.96 (0.84–1.09)	753	1.01 (0.89–1.15)	632	0.97 (0.84–1.11)	0.950	
Liver cancer	311	1	469	0.96 (0.82–1.13)	455	1.09 (0.92–1.29)	331	0.91 (0.76–1.10)	0.683	
All other non-digestive cancers	554	1	892	0.97 (0.86–1.08)	855	1.01 (0.89–1.14)	756	1.00 (0.88–1.14)	0.757	
COPD	207	1	429	1.05 (0.86–1.29)	428	0.97 (0.79–1.20)	288	1.03 (0.82–1.28)	0.897	
All respiratory diseases	302	1	607	1.20 (1.01–1.42)	539	1.06 (0.89–1.26)	395	1.13 (0.94–1.36)	0.591	
Other major chronic diseases	405	1	624	0.98 (0.85–1.14)	570	1.00 (0.86–1.17)	387	0.87 (0.74–1.03)	0.182	
Transport accidents	149	1	333	0.98 (0.80–1.21)	315	1.00 (0.80–1.25)	204	0.87 (0.68–1.11)	0.305	
All other causes	475	1	857	1.03 (0.91–1.17)	723	1.03 (0.89–1.18)	424	0.97 (0.83–1.14)	0.741	

*CI* Confidence interval, *COPD* Chronic obstructive pulmonary disease, *CVD* Cardiovascular disease, *HR* Hazard ratio

^a^Analyses were stratified by age-at-risk (5-year intervals), sex, and study area (10 regions) and adjusted for marital status (never married, married, divorced/separated, or widowed), body mass index (in kg/m^2^; continuous), household income (in yuan/year; <10,000, 10,000–19,999, 20,000–34,999, or ≥35,000), survey season, highest education level (no formal school, primary school, middle or high school, or college and above), physical activity (MET-h/wk), smoking (non-smoker, occasional smoker, ever-smoker, or current smoker), alcohol drinking (non-drinker, occasional drinker, ever-drinker, or current drinker), family history of cancer (yes or no), family history of CVD (yes or no), intakes of red meat, poultry, eggs, fish, soybeans, fruit, dairy (never/rarely, monthly, 1–3 days/week, or regularly), and fresh vegetables (daily or less than daily) at baseline

The level of preserved vegetable consumption was not significantly associated with risk of cancer mortality (*P* = 0.277 for trend) after fully adjusting for socio-economic, demographic, and dietary factors (model 3) (Table 2). However, consumption of preserved vegetables was associated with a higher risk of digestive tract cancer mortality, which was mainly driven by death from esophageal cancer. The multivariable-adjusted HRs (95% CIs) of digestive tract cancer mortality compared with non-consumers were 1.03 (0.92–1.15) for monthly consumers, 1.06 (0.94–1.20) for 1–3 days/week and 1.13 (1.00–1.28) for regular consumers (≥4 days/week) (*P* = 0.053 for trend and *P* = 0.879 for non-linearity). Participants with regular consumption of preserved vegetables had 45% (17–81%) higher risk of mortality from esophageal cancer (*P* = 0.002 for trend). However, preserved vegetable consumption was not significantly associated with deaths from colorectal cancer, lung cancer, liver cancer, or all other non-digestive cancers. No significant association was observed for mortality from respiratory diseases and other major chronic diseases (Table 3).

### Subgroup analyses

The positive association of preserved vegetable consumption with CVD mortality appeared to be stronger among older than younger people (*P* < 0.001 for interaction) and more pronounced among rural than urban residents (*P* = 0.088 for interaction) (Additional file 1: Table S2). The documented relationships did not differ significantly by sex, BMI, smoking, alcohol drinking, physical activity, household income, and blood pressure (*P* > 0.05 for interaction) (Additional file 1: Table S2).

### Sensitivity analyses

Our main findings (positive relationships for hemorrhagic stroke mortality and esophageal cancer) were not substantially changed after adjusting for the dose of cigarettes for current smokers and the dose of alcohol for current drinkers, additionally adjusting for the uses of vitamins and medication (anti-hypertensive and lipid-lowering medication), history of hypertension, self-rated poor health, and a healthy diet score. Excluding participants with extreme BMI and deaths within the initial 2 years of follow-up also did not materially affect our main results (Additional file 1: Tables S3 and S4).

## Discussion

In this large prospective cohort study of more than 0.4 million participants in China, consumption of preserved vegetables was marginally associated with higher risk of CVD mortality. For specific causes of death, preserved vegetable consumption was associated with higher hemorrhagic stroke mortality and higher mortality from digestive tract cancer, especially esophageal cancer. In addition, preserved vegetable consumption was related to increased BMI and blood pressure at baseline.

A previous Chinese Longitudinal Healthy Longevity Survey of 8959 participants showed that occasionally or daily intake of salt-preserved vegetables was positively associated with a 10% higher all-cause mortality among oldest elders (≥80 years) [7]. Here, we found no significant relationship for total mortality but a marginally increased risk of death from CVD, which was mainly driven by increased risk from hemorrhagic stroke mortality. For putative mechanisms, a high level of sodium in salt-preserved vegetables was considered as the main driving factor [23] and we observed 21.6% (12.5–34.7%) of increased CVD mortality being driven by high blood pressure. Previous meta-analyses of randomized trials and prospective studies also have shown that lower sodium intake was associated with lower risk of CVD outcomes including stroke and CHD [24, 25]. For example, a dose response to salt reduction was demonstrated in a meta-analysis of randomized longer-term salt reduction trials, which showed a reduction of 3 g/day would reduce strokes by 13% [25]. A Japan National Integrated Project for Prospective Observation of Non-communicable Disease And its Trends in the Aged in the 1980 National Cardiovascular Survey (NIPPON DATA80) reported that dietary sodium and potassium (Na–K) ratio was significantly related to higher mortality from all-cause stroke (HR: 1.85; 95% CI: 1.22 to 2.83; *P* = 0.002) and CVD (HR: 1.47; 95% CI: 1.10 to 1.96; *P* = 0.005) [26]. They also observed higher mortality from hemorrhagic stroke (HR: 2.34; 95% CI: 1.06 to 5.18; *P* = 0.024) but not ischemic stroke (HR: 1.57; 95% CI: 0.89 to 2.78; *P* = 0.099) [26], which was consistent with our finding. Compared with ischemic stroke, hemorrhagic stroke has been reported to be more strongly related to blood pressure [27]. Here, we found preserved vegetable consumption was dose-dependently related to higher blood pressure. Therefore, preserved vegetable consumption may lead to higher blood pressure and consequently to higher risk of hemorrhagic stroke. In addition, adverse changes in nutritional composition during the process of preservation, including reduction of antioxidants, vitamins, and minerals, may offset potential benefits of fresh vegetables [28]. Notably, pickled and salt-preserved foods are commonly contaminated with N-nitroso compounds due to the conversion from nitrate into nitrite by the process of microbial degradation [29]. A study conducted among the middle east Caucasian residents in Tehran showed that each unit increase in Ln-transformed serum nitrate/nitrite levels was associated with a 35% increase in CVD risk [30]. However, our documented relationship for CVD mortality was overall weak and should be interpreted with caution. Our observed strong detrimental relationship for hemorrhagic stroke was inconsistent with a previous meta-analysis of prospective studies which suggested that pickled vegetable intake was inversely associated with all-cause stroke risk [31]. However, this meta-analysis only included one Chinese study conducted in Jiangxi Province and confounding brought by economic status and seasonal variation for Chinese pickles intake likely explained the finding [32].

Several studies focusing on cancer incidence showed that preserved vegetable consumption was positively associated with colorectal cancer, lung cancer, and kidney cancer [33–35]. Early evidence revealed that the consumption of salt-preserved vegetables was positively related to brain tumor mortality [36]. A recent meta-analysis of cohort studies reported a 15% (7–23%) higher risk of gastric cancer associated with 40 g/day increment in pickled vegetable intake in a dose-response manner [4]. In addition, other case–control studies also revealed a detrimental role of preserved vegetable consumption in developing ESCC [5, 6]. Similar findings were also reported for the association of the processed food pattern, which had high loadings of pickled vegetables, preserved vegetables, salted meat, and salted eggs, with ESCC risk [37]. These data supported our main finding of the elevated risk for death from digestive tract cancer, especially esophageal cancer. For putative mechanisms, a high concentration of salt may induce mucosal damage in the esophagus, followed by cell proliferation and DNA synthesis of damaged cells as part of the repair process, thereby increasing susceptibility to mutagenesis or carcinogenesis [38]. The high content of carcinogenic N-nitroso compounds in processed foods [39, 40] is proposed as another possible mechanism of esophageal carcinogenesis. Nitrosamines as a representative group of N-nitroso compounds are endogenously generated from nitrate and nitrite [41]. Two important nitrosamines, N-nitrosodiethylamine (NDEA) and N-nitrosodimethylamine (NDMA), are classified as probably carcinogenic to humans (group 2A) by the International Agency for Research on Cancer [42]. Nitrosamines may enhance cancer progression by upregulating the expression of cyclinE 1, cyclinD 1, transform growth factor α, and epidermal growth factor receptor in esophageal tissues [43]. Consistent with our results, consuming preserved vegetables was associated with a higher risk of death among ESCC patients in one high-risk area in China [44]. However, the consumption of pickled vegetables preserved by soaking in vinegar or brine and then fermenting in a concealed container for at least 2 weeks was not associated with higher risk of esophageal cancer [41]. The discrepancy of the associations for the consumption of high salty food and pickled vegetables may be due to different preserved methods and the use of condiments other than salt. Pickled vegetable is often soaked by vinegar in a sealed container and thus produces a large amount of *Lactobacillus*, which was associated with a healthier intestinal microenvironment [45], while intestinal *Lactobacillus* was diminished in patients with colorectal cancer [46]. The benefits of *Lactobacillus* and its production may offset the detrimental effect of preserved vegetable consumption on risk of cancer incidence and/or mortality. Differently, Fuling (a district of Chongqing) preserved vegetables, popular in Sichuan province of China, were made by salting fresh cabbages with more than ten kinds of condiments (e.g., cassia bark, pepper, liquorice, and white spirit). In our study, HRs of CVD mortality for ≥4 days/week of preserved vegetable intake seemed lower than those of 1–3 days/week, which could be due to the fact that regular consumers are more aware of pickling vegetables healthily, such as controlling the production of nitrites while increasing beneficial *Lactobacillus.* Future studies are warranted to fully elucidate the causal associations of preserved vegetable consumption with hemorrhagic stroke and esophageal cancer mortality, such as the identification of blood or urinary biomarkers for specifically indicating the negative effects of preserved vegetable consumption on the incidence of these cause-specific mortality risks.

We observed the positive associations were more pronounced among older people and rural residents. It is worth noting that participants who were older or lived in rural areas might preserve vegetables more traditionally, which could be unhealthier, such as Fuling preserved vegetables salted with more than ten kinds of condiments.

The current study is strengthened by the large population size, relatively long follow-up duration, and the large number of deaths from various causes. To our knowledge, it is the first study to examine the association between preserved vegetable consumption and cause-specific mortality. Competing risk from various deaths and adjustment with important non-dietary factors (i.e., lifestyle factors and annual household income) as well as dietary factors (i.e., intakes of fresh vegetables, poultry, and red meat) were also taken into consideration. Furthermore, a series of sensitivity analyses with minimal changes verified the robustness of our findings.

Our study has some limitations. Firstly, the reproducibility of the qualitative FFQ has been validated but we did not estimate the amount of intake in our analysis because only a small subsample of participants (5–6%) reported the amount in the second resurvey. We were not able to check the agreement between self-reported and actual preserved vegetable consumption. Nonetheless, the predictive validity of our preserved vegetable (not nutrient) consumption measures can be inferred from the documented strong associations of preserved vegetable intake with blood pressure and mortality. Secondly, we did not collect information on types, recipes, or preserving techniques of preserved vegetables such as pickled vegetables and salt-preserved vegetables, which is likely to vary greatly for the associations with all-cause and cause-specific mortality due to distinct processing methods. Ingredients including salt, sugar, vinegar, chili peppers, ginger, and garlic could also have differential health effects. Thirdly, the information of salt intake was not collected, which would have provided more insights into the detrimental effect of preserved vegetables on cardiovascular health. Fourthly, although we have performed multivariable-adjusted models and sensitivity analyses with the consideration of various potential confounders, residual confounding, e.g., socio-economic status and other preserved foods, could not be ruled out. Information about total energy intake was not collected. However, the models were adjusted for BMI and total physical activity which can be considered as a proxy of energy balance when assessing the associations of diet and mortality risk. Lastly, a causal association cannot be demonstrated considering the observational nature of the current study.

## Conclusions

Among Chinese population, preserved vegetable intake was marginally associated with higher risk of CVD mortality. Our findings indicated that frequent preserved vegetable consumption might be a dietary risk factor for premature death from hemorrhagic stroke and esophageal cancer. Given the popularity of salt-processed vegetables and pickled vegetables among the Chinese population, limiting the intake of preserved vegetables might confer benefits on these diseases and longevity. However, future studies such as feeding trials are needed to unravel the causal effects of preserved vegetables prepared by different processing methods on health.

## Supplementary Information

**Additional file 1: Table S1.** ICD-10 codes and distribution of deaths at 30-79 years in men and women in the China Kadoorie Biobank. **Table S2.** Adjusted HRs (95% CIs) of subgroup analyses for associations between preserved vegetable consumption and CVD mortality in the China Kadoorie Biobank. **Table S3.** Adjusted HRs (95% CIs) of sensitivity analyses for associations between preserved vegetable consumption and total, CVD and cancer mortality in the China Kadoorie Biobank. **Table S4.** Adjusted HRs (95% CIs) of sensitivity analyses for associations between preserved vegetable consumption and cause-specific mortality in the China Kadoorie Biobank.

## Data Availability

The China Kadoorie Biobank (CKB) is a global resource for the investigation of lifestyle, environmental, blood biochemical, and genetic factors as determinants of common diseases. The CKB study group is committed to making the cohort data available to the scientific community in China, the UK, and worldwide to advance knowledge about the causes, prevention, and treatment of disease. For detailed information on what data is currently available to open access users and how to apply for it, visit: http://www.ckbiobank.org/site/Data+Access. Researchers who are interested in obtaining the raw data from the China Kadoorie Biobank study that underlines this paper should contact ckbaccess@ndph.ox.ac.uk. A research proposal will be requested to ensure that any analysis is performed by bona fide researchers and—where data is not currently available to open access researchers—is restricted to the topic covered in this paper.

## Abbreviations

BMI: Body mass index
CHD: Coronary heart disease
CI: Confidence interval
CKB: China Kadoorie Biobank
CVD: Cardiovascular disease
DBP: Diastolic blood pressure
ESCC: Esophageal squamous cell carcinoma
FFQ: Food frequency questionnaire
HR: Hazard ratio
IHD: Ischemic heart disease
MET: Metabolic equivalent task
NDMA: N-Nitrosodimethylamine
SBP: Systolic blood pressure
