# Supplementary material for: Preserved vegetable consumption and its association with mortality among 440,415 people in the China Kadoorie Biobank
Source: BMC Med. 2023 Apr 5;21:135. doi: 10.1186/s12916-023-02829-3 (PMC10077626; doi:10.1186/s12916-023-02829-3)
Supplement: Supplementary file 1 — Additional file 1: Table S1. ICD-10 codes and distribution of deaths at 30-79 years in men and women in the China Kadoorie Biobank. Table S2. Adjusted HRs (95% CIs) of subgroup analyses for associations between preserved vegetable consumption and CVD mortality in the China Kadoorie Biobank. Table S3. Adjusted HRs (95% CIs) of sensitivity analyses for associations between preserved vegetable consumption and total, CVD and cancer mortality in the China Kadoorie Biobank. Table S4. Adjusted HRs (95% CIs) of sensitivity analyses for associations between preserved vegetable consumption and cause-specific mortality in the China Kadoorie Biobank. [file 12916_2023_2829_MOESM1_ESM.doc]

**ONLINE SUPPLEMENTAL MATERIAL**

**Table of Content**

[Table S1. ICD-10 codes and distribution of deaths at 30-79 years in men and women in the China Kadoorie Biobank 2](#__RefHeading___Toc123656231)

[Table S2. Adjusted HRs (95% CIs) of subgroup analyses for associations between preserved vegetable consumption and CVD mortality in the China Kadoorie Biobank 4](#__RefHeading___Toc123656232)

[Table S3. Adjusted HRs (95% CIs) of sensitivity analyses for associations between preserved vegetable consumption and total, CVD and cancer mortality in the China Kadoorie Biobank 6](#__RefHeading___Toc123656233)

[Table S4. Adjusted HRs (95% CIs) of sensitivity analyses for associations between preserved vegetable consumption and cause-specific mortality in the China Kadoorie Biobank 8](#__RefHeading___Toc123656234)

# Table S1. ICD-10 codes and distribution of deaths at 30-79 years in men and women in the China Kadoorie Biobank

| **Causes of death** | **ICD-10 codes** | **Men (*n* = 177,478)** | |  | **Women (*n* = 262,937)** | |  | **Total (*n* = 440,415)** | |
| --- | --- | --- | --- | --- | --- | --- | --- | --- | --- |
|  |  | **N** | **% death** |  | **N** | **% death** |  | **N** | **% death** |
| Ischemic heart disease | I20-I25 | 2,177 | 13.5% |  | 1,771 | 14.2% |  | 3,948 | 13.8% |
| Ischemic stroke | I63 | 683 | 4.2% |  | 513 | 4.1% |  | 1,196 | 4.2% |
| Haemorrhagic stroke | I61 | 1,906 | 11.8% |  | 1,636 | 13.1% |  | 3,542 | 12.4% |
| Other CVDs | I00-I15, I28-I60, I62, I64-I88, I95-I99 | 1,172 | 7.3% |  | 1,066 | 8.6% |  | 2,238 | 7.8% |
| **All CVDs** | I00-I25, I28-I88, I95-I99 | 5,938 | 36.7% |  | 4,986 | 40.0% |  | 10,924 | 38.2% |
|  |  |  |  |  |  |  |  |  |  |
| **Digestive tract cancer** | C15, C16, C18-C20 | 2,037 | 12.6% |  | 1,106 | 8.9% |  | 3,143 | 11.0% |
| Stomach cancer | C16 | 929 | 5.7% |  | 438 | 3.5% |  | 1,367 | 4.8% |
| Oesophageal cancer | C15 | 727 | 4.5% |  | 290 | 2.3% |  | 1,017 | 3.6% |
| Colorectal cancer | C18-C20 | 381 | 2.4% |  | 378 | 3.0% |  | 759 | 2.7% |
| **Non-digestive tract cancer** | C00-C14, C17, C21-C97 | 3,987 | 24.7% |  | 3,262 | 26.2% |  | 7,249 | 25.3% |
| Lung cancer | C33-C34 | 1,627 | 10.1% |  | 999 | 8.0% |  | 2,626 | 9.2% |
| Liver cancer | C22 | 1,044 | 6.5% |  | 522 | 4.2% |  | 1,566 | 5.5% |
| All other cancers | C00-C14, C17, C21, C23-C32, C35-C97 | 1,316 | 8.1% |  | 1741 | 14.0% |  | 3,057 | 10.7% |
| **All cancer** | C00-C97 | 6,024 | 37.3% |  | 4,368 | 35.0% |  | 10,392 | 36.3% |
|  |  |  |  |  |  |  |  |  |  |
| COPD | J41-J44, I26-I27 | 786 | 4.9% |  | 566 | 4.5% |  | 1,352 | 4.7% |
| **All respiratory diseases** | J00-J99, I26-I27 | 1,076 | 6.7% |  | 767 | 6.2% |  | 1,843 | 6.4% |
| **Other major chronic diseases** | D00-H95, K00-N99 | 1,042 | 6.4% |  | 944 | 7.6% |  | 1,986 | 6.9% |
| **All other causes** | A00-B99, O00-Y99 | 1,450 | 9.0% |  | 1,029 | 8.3% |  | 2,479 | 8.7% |
| Transport accidents | V01-V99 | 631 | 3.9% |  | 370 | 3.0% |  | 1,001 | 3.5% |
|  |  |  |  |  |  |  |  |  |  |
| **Any** |  | 16,161 | 100.0% |  | 12,464 | 100.0% |  | 28,625 | 100.0% |

CVD, cardiovascular disease; COPD, chronic obstructive pulmonary disease

Table S2. Adjusted HRs (95% CIs) of subgroup analyses for associations between preserved vegetable consumption and CVD mortality in the China Kadoorie Biobank**^a^**

|  |  |  | **Preserved vegetable consumption** | | | | ***P* Trend** | ***P* interaction** |
| --- | --- | --- | --- | --- | --- | --- | --- | --- |
| **Subgroups** |  |  | **Never/rarely** | **Monthly** | **1-3 days/week** | **Regularly** |  |  |
|  | **Events** | **N** |  |  |  | **(≥4 days/week)** |  |  |
| **Age** |  |  |  |  |  |  |  | <0.001 |
| <60 years | 3,510 | 351,986 | 1 | 1.02 (0.92-1.13) | 1.08 (0.96-1.21) | 1.06 (0.94-1.20) | 0.223 |  |
| ≥60 years | 7,414 | 88,429 | 1 | 1.11 (1.02-1.20) | 1.15 (1.05-1.25) | 1.07 (0.97-1.17) | 0.121 |  |
| **Sex** |  |  |  |  |  |  |  | 0.537 |
| Men | 5,938 | 177,478 | 1 | 1.13 (1.04-1.22) | 1.12 (1.02-1.23) | 1.06 (0.95-1.17) | 0.307 |  |
| Women | 4,986 | 262,937 | 1 | 1.02 (0.92-1.12) | 1.13 (1.02-1.25) | 1.09 (0.97-1.21) | 0.054 |  |
| **BMI** |  |  |  |  |  |  |  | 0.192 |
| <24 | 6,536 | 251,759 | 1 | 1.11 (1.02-1.21) | 1.15 (1.05-1.26) | 1.06 (0.96-1.18) | 0.191 |  |
| ≥24 | 4,388 | 188,656 | 1 | 1.04 (0.94-1.14) | 1.08 (0.98-1.20) | 1.07 (0.96-1.20) | 0.164 |  |
| **Study area** |  |  |  |  |  |  |  | 0.088 |
| Rural | 7,474 | 250,393 | 1 | 1.07 (0.99-1.16) | 1.16 (1.07-1.27) | 1.12 (1.01-1.24) | 0.007 |  |
| Urban | 3,450 | 190,022 | 1 | 1.11 (0.99-1.23) | 1.04 (0.93-1.17) | 1.01 (0.90-1.13) | 0.929 |  |
| **Smoking** |  |  |  |  |  |  |  | 0.279 |
| Non/former smoker | 7,057 | 323,792 | 1 | 1.06 (0.98-1.14) | 1.16 (1.07-1.27) | 1.09 (0.99-1.19) | 0.021 |  |
| Current smoker | 3,867 | 116,623 | 1 | 1.11 (0.99-1.23) | 1.06 (0.94-1.19) | 1.04 (0.91-1.17) | 0.873 |  |
| **Alcohol drinking** |  |  |  |  |  |  |  | 0.204 |
| Nondrinker | 8,660 | 349,326 | 1 | 1.07 (1.00-1.15) | 1.14 (1.05-1.23) | 1.06 (0.97-1.15) | 0.092 |  |
| Drinker | 2,264 | 91,089 | 1 | 1.08 (0.94-1.24) | 1.03 (0.89-1.20) | 1.11 (0.95-1.30) | 0.290 |  |
| **Physical activity** |  |  |  |  |  |  |  | 0.467 |
| <Median | 7,938 | 220,197 | 1 | 1.08 (1.00-1.15) | 1.10 (1.01-1.19) | 1.09 (0.99-1.19) | 0.047 |  |
| ≥Median | 2,986 | 220,218 | 1 | 1.05 (0.92-1.21) | 1.16 (1.01-1.34) | 1.06 (0.91-1.23) | 0.369 |  |
| **Household income** |  |  |  |  |  |  |  | 0.389 |
| <20,000 | 8,303 | 249,287 | 1 | 1.07 (1.00-1.15) | 1.10 (1.01-1.19) | 1.06 (0.97-1.16) | 0.151 |  |
| ≥20,000 | 2,621 | 191,128 | 1 | 1.09 (0.96-1.25) | 1.20 (1.04-1.38) | 1.09 (0.93-1.28) | 0.090 |  |
| **Blood pressure^b^** |  |  |  |  |  |  |  | 0.699 |
| Non-hypertensive | 3,522 | 302,041 | 1 | 1.16 (1.04-1.29) | 1.11 (0.98-1.25) | 1.12 (0.98-1.28) | 0.176 |  |
| Hypertensive | 7,402 | 138,374 | 1 | 1.02 (0.94-1.10) | 1.11 (1.02-1.20) | 1.01 (0.92-1.10) | 0.453 |  |

BMI, body mass index; CI, confidence interval; CVD, cardiovascular disease; HR, hazard ratio.

^a^The multivariate-adjusted model was stratified by age-at-risk (5-year intervals), sex and study area (10 regions), adjusted for marital status (never married, married, divorced/separated, or widowed), body mass index (in kg/m^2^; continuous), household income (in yuan/year; <10,000, 10,000-19,999, 20,000-34,999, or ≥35,000), survey season, highest education level (no formal school, primary school, middle or high school, or college and above), physical activity (MET-h/wk), smoking (non-smoker, occasional smoker, ever-smoker, or current smoker), alcohol drinking (non-drinker, occasional drinker, ever-drinker, or current drinker), family history of cancer (yes or no), family history of CVD (yes or no), intake of red meat, poultry, eggs, fish, soybeans, fruit, dairy (never/rarely, monthly, 1-3 days/week, regularly) and fresh vegetables (daily or less than daily) at baseline.

^b^Hypertension was defined as SBP ≥ 140 mm Hg, DBP ≥ 90 mm Hg, having known hypertension, or current hypotensive drugs use.

# Table S3. Adjusted HRs (95% CIs) of sensitivity analyses for associations between preserved vegetable consumption and total, CVD and cancer mortality in the China Kadoorie Biobank^a^

|  | **Events** | **Preserved vegetable consumption** | | | |  |
| --- | --- | --- | --- | --- | --- | --- |
|  |  | **Never/rarely** | **Monthly** | **1-3 days/week** | **Regularly** | ***P* Trend** |
|  |  |  |  |  | **(≥4 days/week)** |  |
| **Excluding extreme BMI (n= 421,796)** | |  |  |  |  |  |
| Total mortality | 26,177 | 1 | 1.03 (0.99-1.07) | 1.06 (1.01-1.10) | 1.02 (0.98-1.07) | 0.183 |
| CVD mortality | 10,050 | 1 | 1.07 (1.01-1.14) | 1.12 (1.04-1.20) | 1.06 (0.98-1.15) | 0.069 |
| Cancer mortality | 9,671 | 1 | 0.99 (0.93-1.05) | 1.05 (0.98-1.12) | 1.04 (0.97-1.11) | 0.143 |
| **Excluding deaths in the initial 2 years of follow-up (n=437,150)** | |  |  |  |  |  |
| Total mortality | 25,444 | 1 | 1.04 (1.00-1.08) | 1.07 (1.03-1.12) | 1.03 (0.98-1.08) | 0.139 |
| CVD mortality | 9,891 | 1 | 1.09 (1.02-1.16) | 1.13 (1.05-1.22) | 1.08 (1.00-1.17) | 0.027 |
| Cancer mortality | 9,194 | 1 | 0.98 (0.92-1.05) | 1.03 (0.96-1.10) | 1.02 (0.94-1.09) | 0.393 |
| **Adjusting for dose of cigarettes and alcohol**^b^ **(n=440,415)** | |  |  |  |  |  |
| Total mortality | 28,625 | 1 | 1.03 (0.99-1.07) | 1.06 (1.02-1.11) | 1.02 (0.97-1.06) | 0.287 |
| CVD mortality | 10,924 | 1 | 1.07 (1.01-1.14) | 1.12 (1.05-1.20) | 1.06 (0.99-1.15) | 0.058 |
| Cancer mortality | 10,392 | 1 | 0.98 (0.92-1.05) | 1.04 (0.97-1.11) | 1.01 (0.95-1.09) | 0.374 |
| **Further adjusted for vitamin use (n=440,415)** |  |  |  |  |  |  |
| Total mortality | 28,625 | 1 | 1.03 (0.99-1.07) | 1.06 (1.02-1.11) | 1.02 (0.98-1.07) | 0.174 |
| CVD mortality | 10,924 | 1 | 1.08 (1.01-1.14) | 1.12 (1.05-1.20) | 1.07 (0.99-1.15) | 0.044 |
| Cancer mortality | 10,392 | 1 | 0.98 (0.93-1.05) | 1.04 (0.97-1.11) | 1.02 (0.95-1.09) | 0.279 |
| **Further adjusted for hypertension (n=440,415)** |  |  |  |  |  |  |
| Total mortality | 28,625 | 1 | 1.03 (0.99-1.07) | 1.06 (1.02-1.11) | 1.02 (0.98-1.07) | 0.184 |
| CVD mortality | 10,924 | 1 | 1.07 (1.01-1.14) | 1.13 (1.05-1.21) | 1.07 (0.99-1.15) | 0.037 |
| Cancer mortality | 10,392 | 1 | 0.98 (0.93-1.05) | 1.04 (0.97-1.11) | 1.02 (0.95-1.10) | 0.277 |
| **Further adjusted for self-rated poor health (n=440,415)** | |  |  |  |  |  |
| Total mortality | 28,625 | 1 | 1.04 (1.00-1.08) | 1.07 (1.03-1.12) | 1.03 (0.98-1.07) | 0.118 |
| CVD mortality | 10,924 | 1 | 1.08 (1.02-1.15) | 1.14 (1.06-1.22) | 1.07 (0.99-1.15) | 0.035 |
| Cancer mortality | 10,392 | 1 | 0.99 (0.93-1.05) | 1.04 (0.98-1.12) | 1.02 (0.95-1.10) | 0.252 |
| **Further adjusted for antihypertensive treatments (n=440,415)** | |  |  |  |  |  |
| Total mortality | 28,625 | 1 | 1.03 (0.99-1.07) | 1.06 (1.02-1.11) | 1.02 (0.98-1.07) | 0.172 |
| CVD mortality | 10,924 | 1 | 1.07 (1.01-1.14) | 1.12 (1.05-1.20) | 1.07 (0.99-1.16) | 0.038 |
| Cancer mortality | 10,392 | 1 | 0.98 (0.93-1.05) | 1.04 (0.97-1.11) | 1.02 (0.95-1.10) | 0.277 |
| **Further adjusted for lipid-lowing treatments (n=440,415)** | |  |  |  |  |  |
| Total mortality | 28,625 | 1 | 1.03 (0.99-1.07) | 1.06 (1.02-1.11) | 1.02 (0.98-1.07) | 0.170 |
| CVD mortality | 10,924 | 1 | 1.08 (1.01-1.15) | 1.12 (1.05-1.20) | 1.07 (0.99-1.15) | 0.041 |
| Cancer mortality | 10,392 | 1 | 0.98 (0.93-1.05) | 1.04 (0.97-1.11) | 1.02 (0.95-1.09) | 0.277 |
| **Further adjusted for healthy diet (n=440,415)** | |  |  |  |  |  |
| Total mortality | 28,625 | 1 | 1.03 (0.99-1.07) | 1.06 (1.01-1.10) | 1.02 (0.97-1.07) | 0.313 |
| CVD mortality | 10,924 | 1 | 1.08 (1.01-1.14) | 1.13 (1.05-1.21) | 1.07 (0.99-1.16) | 0.048 |
| Cancer mortality | 10,392 | 1 | 0.99 (0.93-1.05) | 1.04 (0.97-1.11) | 1.02 (0.95-1.09) | 0.400 |

BMI, body mass index; CI, confidence interval; CVD, cardiovascular disease; HR, hazard ratio.

^a^The multivariate-adjusted model was stratified by age-at-risk (5-year intervals), sex and study area (10 regions), body mass index (in kg/m^2^; continuous), household income (in yuan/year; <10,000, 10,000-19,999, 20,000-34,999, or ≥35,000), survey season, highest education level (no formal school, primary school, middle or high school, or college and above), physical activity (MET-h/wk), smoking (non-smoker, occasional smoker, ever-smoker, or current smoker), alcohol drinking (non-drinker, occasional drinker, ever-drinker, or current drinker), family history of cancer (yes or no), family history of CVD (yes or no), intake of red meat, poultry, eggs, fish, soybeans, fruit, dairy (never/rarely, monthly, 1-3 days/week, regularly) and fresh vegetables (daily or less than daily) at baseline.

^b^Dose of cigarettes for current smokers [<20, 20-24, and ≥25 cigarettes (or equivalent) per day] and dose of alcohol for current drinkers (<140, 140 to <420, and ≥420 g of alcohol per week) were controlled.

# Table S4. Adjusted HRs (95% CIs) of sensitivity analyses for associations between preserved vegetable consumption and cause-specific mortality in the China Kadoorie Biobank^a^

|  | **Events** | **Preserved vegetable consumption** | | | | ***P* Trend** |
| --- | --- | --- | --- | --- | --- | --- |
|  |  | **Never/rarely** | **Monthly** | **1-3 days/week** | **Regularly** |  |
|  |  |  |  |  | **(≥4 days/week)** |  |
| **Excluding extreme BMI (n= 421,796)** | |  |  |  |  |  |
| Ischemic heart disease | 3,620 | 1 | 1.06 (0.95-1.17) | 1.02 (0.91-1.15) | 1.06 (0.94-1.20) | 0.481 |
| Ischemic stroke | 1,093 | 1 | 1.03 (0.85-1.25) | 0.96 (0.77-1.20) | 1.00 (0.79-1.27) | 0.885 |
| Haemorrhagic stroke | 3,265 | 1 | 1.08 (0.95-1.22) | 1.31 (1.15-1.49) | 1.13 (0.98-1.30) | 0.012 |
| Other CVDs | 2,072 | 1 | 1.13 (0.99-1.30) | 1.06 (0.91-1.24) | 1.02 (0.86-1.22) | 0.894 |
| Stomach cancer | 1,246 | 1 | 1.01 (0.83-1.21) | 1.10 (0.90-1.34) | 1.10 (0.90-1.34) | 0.280 |
| Oesophageal cancer | 943 | 1 | 0.93 (0.78-1.12) | 0.94 (0.74-1.20) | 1.43 (1.14-1.79) | 0.005 |
| Colorectal cancer | 713 | 1 | 1.24 (0.96-1.59) | 1.15 (0.89-1.49) | 1.00 (0.76-1.31) | 0.681 |
| Digestive tract cancer | 2,902 | 1 | 1.02 (0.91-1.15) | 1.06 (0.93-1.20) | 1.14 (1.01-1.30) | 0.038 |
| Non-digestive tract cancer | 6,769 | 1 | 0.97 (0.90-1.05) | 1.04 (0.96-1.13) | 0.99 (0.91-1.08) | 0.709 |
| Lung cancer | 2,401 | 1 | 0.96 (0.84-1.10) | 1.03 (0.90-1.19) | 1.00 (0.86-1.15) | 0.692 |
| Liver cancer | 1,478 | 1 | 0.96 (0.82-1.13) | 1.09 (0.92-1.30) | 0.91 (0.75-1.10) | 0.651 |
| All other non-digestive cancers | 2,890 | 1 | 0.99 (0.88-1.11) | 1.02 (0.90-1.16) | 1.02 (0.90-1.16) | 0.620 |
| COPD | 1,037 | 1 | 1.10 (0.88-1.39) | 0.95 (0.75-1.20) | 1.09 (0.85-1.40) | 0.892 |
| All respiratory diseases | 1,471 | 1 | 1.23 (1.02-1.49) | 1.03 (0.85-1.26) | 1.17 (0.95-1.44) | 0.501 |
| Other major chronic diseases | 1,817 | 1 | 0.96 (0.83-1.11) | 0.95 (0.81-1.12) | 0.85 (0.71-1.01) | 0.088 |
| Transport accidents | 934 | 1 | 0.96 (0.77-1.19) | 0.97 (0.77-1.22) | 0.84 (0.65-1.08) | 0.220 |
| All other causes | 2,234 | 1 | 1.02 (0.89-1.16) | 1.02 (0.88-1.17) | 0.95 (0.81-1.12) | 0.614 |
| **Excluding deaths in the initial 2 years of follow-up (n=437,150)** | | |  |  |  |  |
| Ischemic heart disease | 3,619 | 1 | 1.10 (0.99-1.22) | 1.03 (0.91-1.16) | 1.11 (0.98-1.26) | 0.264 |
| Ischemic stroke | 1,109 | 1 | 1.03 (0.84-1.25) | 1.03 (0.82-1.28) | 1.01 (0.79-1.28) | 0.957 |
| Haemorrhagic stroke | 3,089 | 1 | 1.12 (0.98-1.28) | 1.37 (1.19-1.56) | 1.18 (1.01-1.37) | 0.004 |
| Other CVDs | 2,074 | 1 | 1.08 (0.94-1.24) | 1.05 (0.90-1.22) | 0.99 (0.83-1.18) | 0.902 |
| Stomach cancer | 1,182 | 1 | 1.00 (0.83-1.22) | 1.10 (0.90-1.34) | 1.06 (0.87-1.30) | 0.453 |
| Oesophageal cancer | 885 | 1 | 0.92 (0.76-1.11) | 0.92 (0.71-1.18) | 1.48 (1.17-1.86) | 0.003 |
| Colorectal cancer | 708 | 1 | 1.23 (0.95-1.59) | 1.15 (0.88-1.49) | 1.02 (0.77-1.34) | 0.803 |
| Digestive tract cancer | 2,775 | 1 | 1.02 (0.90-1.14) | 1.05 (0.92-1.20) | 1.14 (1.00-1.30) | 0.047 |
| Non-digestive tract cancer | 6,419 | 1 | 0.96 (0.89-1.04) | 1.01 (0.93-1.10) | 0.96 (0.88-1.05) | 0.760 |
| Lung cancer | 2,366 | 1 | 0.97 (0.85-1.11) | 1.01 (0.88-1.16) | 0.99 (0.85-1.15) | 0.926 |
| Liver cancer | 1,320 | 1 | 0.96 (0.81-1.13) | 1.07 (0.89-1.28) | 0.87 (0.71-1.06) | 0.383 |
| All other non-digestive cancers | 2,733 | 1 | 0.96 (0.85-1.08) | 0.99 (0.87-1.12) | 0.99 (0.86-1.13) | 0.989 |
| COPD | 1,192 | 1 | 1.10 (0.88-1.37) | 0.99 (0.79-1.25) | 1.09 (0.86-1.39) | 0.812 |
| All respiratory diseases | 1,656 | 1 | 1.25 (1.04-1.49) | 1.10 (0.91-1.33) | 1.20 (0.98-1.46) | 0.327 |
| Other major chronic diseases | 1,775 | 1 | 0.98 (0.84-1.14) | 1.03 (0.87-1.21) | 0.86 (0.71-1.03) | 0.192 |
| Transport accidents | 815 | 1 | 0.97 (0.76-1.22) | 1.05 (0.82-1.35) | 0.77 (0.58-1.01) | 0.141 |
| All other causes | 2,113 | 1 | 1.03 (0.90-1.18) | 1.05 (0.90-1.22) | 0.98 (0.83-1.17) | 0.971 |
| **Adjusting for dose of cigarettes and alcohol**^b^ **(n=440,415)** | |  |  |  |  |  |
| Ischemic heart disease | 3,948 | 1 | 1.06 (0.96-1.18) | 1.00 (0.89-1.12) | 1.07 (0.95-1.21) | 0.453 |
| Ischemic stroke | 1,196 | 1 | 1.04 (0.86-1.25) | 1.01 (0.82-1.25) | 1.02 (0.81-1.28) | 0.954 |
| Haemorrhagic stroke | 3,542 | 1 | 1.09 (0.96-1.23) | 1.32 (1.16-1.49) | 1.13 (0.99-1.30) | 0.009 |
| Other CVDs | 2,238 | 1 | 1.11 (0.97-1.26) | 1.08 (0.93-1.26) | 0.99 (0.84-1.17) | 0.965 |
| Stomach cancer | 1,367 | 1 | 1.00 (0.83-1.19) | 1.05 (0.87-1.27) | 1.05 (0.87-1.26) | 0.558 |
| Oesophageal cancer | 1,017 | 1 | 0.96 (0.81-1.15) | 0.97 (0.77-1.23) | 1.40 (1.13-1.75) | 0.006 |
| Colorectal cancer | 759 | 1 | 1.24 (0.97-1.58) | 1.18 (0.91-1.51) | 1.00 (0.77-1.31) | 0.751 |
| Digestive tract cancer | 3,143 | 1 | 1.03 (0.92-1.15) | 1.06 (0.93-1.20) | 1.12 (0.99-1.27) | 0.081 |
| Non-digestive tract cancer | 7,249 | 1 | 0.96 (0.89-1.04) | 1.03 (0.95-1.11) | 0.97 (0.89-1.05) | 0.903 |
| Lung cancer | 2,626 | 1 | 0.95 (0.84-1.08) | 1.01 (0.88-1.15) | 0.97 (0.84-1.11) | 0.911 |
| Liver cancer | 1,566 | 1 | 0.96 (0.82-1.12) | 1.09 (0.92-1.29) | 0.90 (0.75-1.09) | 0.611 |
| All other non-digestive cancers | 3,057 | 1 | 0.96 (0.86-1.08) | 1.01 (0.89-1.14) | 1.00 (0.88-1.13) | 0.829 |
| COPD | 1,352 | 1 | 1.05 (0.86-1.29) | 0.97 (0.78-1.19) | 1.02 (0.82-1.28) | 0.870 |
| All respiratory diseases | 1,843 | 1 | 1.20 (1.01-1.42) | 1.05 (0.88-1.26) | 1.12 (0.93-1.35) | 0.638 |
| Other major chronic diseases | 1,986 | 1 | 0.98 (0.85-1.13) | 1.00 (0.86-1.17) | 0.87 (0.73-1.03) | 0.155 |
| Transport accidents | 1,001 | 1 | 0.98 (0.79-1.21) | 1.00 (0.80-1.25) | 0.86 (0.67-1.10) | 0.286 |
| All other causes | 2,479 | 1 | 1.03 (0.91-1.17) | 1.02 (0.89-1.18) | 0.96 (0.82-1.13) | 0.642 |
| **Further adjusted for vitamin use (n=440415)** | |  |  |  |  |  |
| Ischemic heart disease | 3,948 | 1 | 1.06 (0.96-1.18) | 1.00 (0.90-1.12) | 1.08 (0.95-1.22) | 0.417 |
| Ischemic stroke | 1,196 | 1 | 1.04 (0.86-1.26) | 1.01 (0.81-1.25) | 1.02 (0.81-1.28) | 0.947 |
| Haemorrhagic stroke | 3,542 | 1 | 1.09 (0.97-1.23) | 1.32 (1.16-1.50) | 1.14 (1.00-1.31) | 0.007 |
| Other CVDs | 2,238 | 1 | 1.11 (0.97-1.26) | 1.08 (0.93-1.26) | 0.99 (0.84-1.17) | 0.993 |
| Stomach cancer | 1,367 | 1 | 0.99 (0.83-1.19) | 1.05 (0.87-1.27) | 1.04 (0.86-1.26) | 0.591 |
| Oesophageal cancer | 1,017 | 1 | 0.96 (0.81-1.14) | 0.98 (0.77-1.23) | 1.46 (1.17-1.81) | 0.002 |
| Colorectal cancer | 759 | 1 | 1.24 (0.97-1.58) | 1.18 (0.91-1.51) | 1.00 (0.77-1.31) | 0.754 |
| Digestive tract cancer | 3,143 | 1 | 1.03 (0.92-1.15) | 1.06 (0.93-1.20) | 1.13 (1.00-1.28) | 0.055 |
| Non-digestive tract cancer | 7,249 | 1 | 0.96 (0.89-1.04) | 1.03 (0.95-1.11) | 0.97 (0.90-1.06) | 0.992 |
| Lung cancer | 2,626 | 1 | 0.96 (0.84-1.09) | 1.01 (0.89-1.15) | 0.97 (0.84-1.11) | 0.949 |
| Liver cancer | 1,566 | 1 | 0.96 (0.82-1.13) | 1.09 (0.92-1.29) | 0.91 (0.76-1.10) | 0.685 |
| All other non-digestive cancers | 3,057 | 1 | 0.97 (0.86-1.08) | 1.01 (0.89-1.14) | 1.00 (0.88-1.14) | 0.749 |
| COPD | 1,352 | 1 | 1.05 (0.86-1.29) | 0.97 (0.79-1.20) | 1.03 (0.82-1.28) | 0.901 |
| All respiratory diseases | 1,843 | 1 | 1.20 (1.01-1.42) | 1.06 (0.89-1.26) | 1.13 (0.94-1.36) | 0.586 |
| Other major chronic diseases | 1,986 | 1 | 0.98 (0.85-1.14) | 1.00 (0.86-1.17) | 0.87 (0.74-1.03) | 0.183 |
| Transport accidents | 1,001 | 1 | 0.98 (0.80-1.21) | 1.00 (0.80-1.25) | 0.87 (0.68-1.11) | 0.302 |
| All other causes | 2,479 | 1 | 1.03 (0.91-1.17) | 1.03 (0.89-1.18) | 0.97 (0.83-1.14) | 0.737 |
| **Further adjusted for hypertension (n=440,415)** | |  |  |  |  |  |
| Ischemic heart disease | 3,948 | 1 | 1.06 (0.96-1.18) | 1.01 (0.90-1.13) | 1.08 (0.96-1.22) | 0.356 |
| Ischemic stroke | 1,196 | 1 | 1.05 (0.87-1.26) | 1.02 (0.82-1.26) | 1.02 (0.81-1.28) | 0.940 |
| Haemorrhagic stroke | 3,542 | 1 | 1.08 (0.96-1.22) | 1.32 (1.17-1.50) | 1.14 (0.99-1.31) | 0.006 |
| Other CVDs | 2,238 | 1 | 1.11 (0.97-1.27) | 1.08 (0.93-1.26) | 0.99 (0.83-1.17) | 0.940 |
| Stomach cancer | 1,367 | 1 | 0.99 (0.83-1.19) | 1.05 (0.87-1.27) | 1.04 (0.86-1.26) | 0.566 |
| Oesophageal cancer | 1,017 | 1 | 0.96 (0.81-1.14) | 0.97 (0.77-1.23) | 1.45 (1.17-1.81) | 0.002 |
| Colorectal cancer | 759 | 1 | 1.24 (0.97-1.58) | 1.18 (0.91-1.51) | 1.00 (0.77-1.31) | 0.755 |
| Digestive tract cancer | 3,143 | 1 | 1.03 (0.92-1.15) | 1.06 (0.94-1.20) | 1.13 (1.00-1.28) | 0.053 |
| Non-digestive tract cancer | 7,249 | 1 | 0.96 (0.89-1.04) | 1.03 (0.95-1.11) | 0.97 (0.90-1.06) | 0.996 |
| Lung cancer | 2,626 | 1 | 0.96 (0.84-1.09) | 1.01 (0.89-1.15) | 0.97 (0.84-1.11) | 0.949 |
| Liver cancer | 1,566 | 1 | 0.96 (0.82-1.13) | 1.09 (0.92-1.29) | 0.91 (0.76-1.10) | 0.686 |
| All other non-digestive cancers | 3,057 | 1 | 0.97 (0.86-1.08) | 1.01 (0.89-1.14) | 1.00 (0.88-1.14) | 0.753 |
| COPD | 1,352 | 1 | 1.05 (0.86-1.29) | 0.97 (0.78-1.19) | 1.02 (0.82-1.28) | 0.877 |
| All respiratory diseases | 1,843 | 1 | 1.20 (1.01-1.41) | 1.06 (0.89-1.26) | 1.13 (0.93-1.36) | 0.609 |
| Other major chronic diseases | 1,986 | 1 | 0.98 (0.85-1.13) | 1.00 (0.86-1.17) | 0.87 (0.73-1.03) | 0.166 |
| Transport accidents | 1,001 | 1 | 0.98 (0.80-1.21) | 1.00 (0.80-1.25) | 0.87 (0.68-1.11) | 0.304 |
| All other causes | 2,479 | 1 | 1.03 (0.91-1.17) | 1.03 (0.89-1.18) | 0.97 (0.83-1.14) | 0.728 |
| **Further adjusted for self-rated poor health** **(n=440,415)** | | |  |  |  |  |
| Ischemic heart disease | 3,948 | 1 | 1.07 (0.97-1.18) | 1.02 (0.91-1.14) | 1.08 (0.96-1.22) | 0.394 |
| Ischemic stroke | 1,196 | 1 | 1.05 (0.87-1.27) | 1.03 (0.83-1.27) | 1.02 (0.81-1.28) | 0.934 |
| Haemorrhagic stroke | 3,542 | 1 | 1.10 (0.98-1.24) | 1.34 (1.18-1.52) | 1.15 (1.00-1.32) | 0.006 |
| Other CVDs | 2,238 | 1 | 1.12 (0.98-1.28) | 1.10 (0.95-1.28) | 1.00 (0.84-1.18) | 0.941 |
| Stomach cancer | 1,367 | 1 | 1.00 (0.83-1.19) | 1.06 (0.88-1.28) | 1.05 (0.87-1.26) | 0.554 |
| Oesophageal cancer | 1,017 | 1 | 0.96 (0.81-1.14) | 0.98 (0.77-1.23) | 1.46 (1.17-1.81) | 0.002 |
| Colorectal cancer | 759 | 1 | 1.24 (0.97-1.58) | 1.18 (0.92-1.52) | 1.00 (0.77-1.31) | 0.768 |
| Digestive tract cancer | 3,143 | 1 | 1.03 (0.92-1.15) | 1.06 (0.94-1.20) | 1.13 (1.00-1.28) | 0.050 |
| Non-digestive tract cancer | 7,249 | 1 | 0.97 (0.90-1.04) | 1.03 (0.96-1.12) | 0.98 (0.90-1.06) | 0.955 |
| Lung cancer | 2,626 | 1 | 0.96 (0.84-1.09) | 1.01 (0.89-1.16) | 0.97 (0.84-1.12) | 0.954 |
| Liver cancer | 1,566 | 1 | 0.97 (0.83-1.13) | 1.10 (0.93-1.31) | 0.92 (0.76-1.10) | 0.723 |
| All other non-digestive cancers | 3,057 | 1 | 0.97 (0.86-1.09) | 1.01 (0.90-1.14) | 1.01 (0.89-1.14) | 0.724 |
| COPD | 1,352 | 1 | 1.08 (0.88-1.32) | 1.00 (0.81-1.23) | 1.04 (0.84-1.30) | 0.987 |
| All respiratory diseases | 1,843 | 1 | 1.21 (1.03-1.44) | 1.08 (0.91-1.29) | 1.14 (0.94-1.37) | 0.518 |
| Other major chronic diseases | 1,986 | 1 | 1.00 (0.87-1.16) | 1.03 (0.88-1.20) | 0.88 (0.75-1.05) | 0.246 |
| Transport accidents | 1,001 | 1 | 0.98 (0.80-1.22) | 1.00 (0.80-1.26) | 0.87 (0.68-1.11) | 0.310 |
| All other causes | 2,479 | 1 | 1.04 (0.92-1.18) | 1.04 (0.90-1.19) | 0.97 (0.83-1.14) | 0.771 |
| **Further adjusted for antihypertensive medicine** **(n=440,415)** | | |  |  |  |  |
| Ischemic heart disease | 3,948 | 1 | 1.06 (0.96-1.18) | 1.01 (0.90-1.13) | 1.08 (0.96-1.22) | 0.383 |
| Ischemic stroke | 1,196 | 1 | 1.04 (0.86-1.26) | 1.01 (0.82-1.25) | 1.02 (0.81-1.28) | 0.949 |
| Haemorrhagic stroke | 3,542 | 1 | 1.08 (0.96-1.22) | 1.32 (1.16-1.49) | 1.15 (1.00-1.32) | 0.005 |
| Other CVDs | 2,238 | 1 | 1.11 (0.97-1.26) | 1.08 (0.93-1.26) | 0.99 (0.84-1.17) | 0.966 |
| Stomach cancer | 1,367 | 1 | 0.99 (0.83-1.19) | 1.05 (0.87-1.27) | 1.04 (0.86-1.26) | 0.564 |
| Oesophageal cancer | 1,017 | 1 | 0.96 (0.81-1.14) | 0.97 (0.77-1.23) | 1.45 (1.17-1.81) | 0.002 |
| Colorectal cancer | 759 | 1 | 1.24 (0.97-1.58) | 1.18 (0.91-1.51) | 1.00 (0.77-1.31) | 0.756 |
| Digestive tract cancer | 3,143 | 1 | 1.03 (0.92-1.15) | 1.06 (0.94-1.20) | 1.13 (1.00-1.28) | 0.052 |
| Non-digestive tract cancer | 7,249 | 1 | 0.96 (0.89-1.04) | 1.03 (0.95-1.11) | 0.97 (0.89-1.06) | 0.999 |
| Lung cancer | 2,626 | 1 | 0.95 (0.84-1.09) | 1.01 (0.89-1.15) | 0.97 (0.84-1.11) | 0.951 |
| Liver cancer | 1,566 | 1 | 0.96 (0.82-1.13) | 1.09 (0.92-1.29) | 0.91 (0.76-1.10) | 0.684 |
| All other non-digestive cancers | 3,057 | 1 | 0.97 (0.86-1.08) | 1.01 (0.89-1.14) | 1.00 (0.88-1.14) | 0.756 |
| COPD | 1,352 | 1 | 1.05 (0.86-1.29) | 0.97 (0.78-1.19) | 1.03 (0.82-1.28) | 0.895 |
| All respiratory diseases | 1,843 | 1 | 1.20 (1.01-1.42) | 1.06 (0.89-1.26) | 1.13 (0.94-1.36) | 0.596 |
| Other major chronic diseases | 1,986 | 1 | 0.98 (0.85-1.13) | 1.00 (0.86-1.17) | 0.87 (0.74-1.03) | 0.179 |
| Transport accidents | 1,001 | 1 | 0.98 (0.80-1.21) | 1.00 (0.80-1.25) | 0.87 (0.68-1.11) | 0.305 |
| All other causes | 2,479 | 1 | 1.03 (0.91-1.17) | 1.03 (0.89-1.18) | 0.97 (0.83-1.14) | 0.739 |
| **Further adjusted for lipid-lowing treatments** **(n=440,415)** | | |  |  |  |  |
| Ischemic heart disease | 3,948 | 1 | 1.06 (0.96-1.18) | 1.01 (0.90-1.13) | 1.08 (0.96-1.22) | 0.401 |
| Ischemic stroke | 1,196 | 1 | 1.04 (0.86-1.26) | 1.01 (0.82-1.25) | 1.02 (0.81-1.28) | 0.940 |
| Haemorrhagic stroke | 3,542 | 1 | 1.09 (0.97-1.23) | 1.32 (1.16-1.50) | 1.15 (1.00-1.31) | 0.006 |
| Other CVDs | 2,238 | 1 | 1.11 (0.97-1.26) | 1.08 (0.93-1.26) | 0.99 (0.84-1.17) | 0.992 |
| Stomach cancer | 1,367 | 1 | 0.99 (0.83-1.19) | 1.05 (0.87-1.27) | 1.04 (0.86-1.26) | 0.566 |
| Oesophageal cancer | 1,017 | 1 | 0.96 (0.81-1.15) | 0.97 (0.77-1.23) | 1.45 (1.17-1.81) | 0.002 |
| Colorectal cancer | 759 | 1 | 1.24 (0.97-1.58) | 1.18 (0.92-1.51) | 1.00 (0.77-1.31) | 0.756 |
| Digestive tract cancer | 3,143 | 1 | 1.03 (0.92-1.15) | 1.06 (0.93-1.20) | 1.13 (1.00-1.28) | 0.053 |
| Non-digestive tract cancer | 7,249 | 1 | 0.96 (0.89-1.04) | 1.03 (0.95-1.11) | 0.97 (0.89-1.06) | 0.997 |
| Lung cancer | 2,626 | 1 | 0.95 (0.84-1.09) | 1.01 (0.89-1.15) | 0.97 (0.84-1.11) | 0.951 |
| Liver cancer | 1,566 | 1 | 0.96 (0.83-1.13) | 1.09 (0.92-1.29) | 0.91 (0.76-1.10) | 0.682 |
| All other non-digestive cancers | 3,057 | 1 | 0.97 (0.86-1.08) | 1.01 (0.89-1.14) | 1.00 (0.88-1.14) | 0.755 |
| COPD | 1,352 | 1 | 1.06 (0.86-1.29) | 0.97 (0.79-1.20) | 1.03 (0.82-1.28) | 0.898 |
| All respiratory diseases | 1,843 | 1 | 1.20 (1.01-1.42) | 1.06 (0.89-1.26) | 1.13 (0.94-1.36) | 0.591 |
| Other major chronic diseases | 1,986 | 1 | 0.98 (0.85-1.14) | 1.00 (0.86-1.17) | 0.87 (0.74-1.03) | 0.184 |
| Transport accidents | 1,001 | 1 | 0.98 (0.80-1.21) | 1.00 (0.80-1.25) | 0.87 (0.68-1.11) | 0.302 |
| All other causes | 2,479 | 1 | 1.03 (0.91-1.17) | 1.03 (0.89-1.18) | 0.97 (0.83-1.14) | 0.741 |
| **Further adjusted for healthy diet** **(n=440,415)** |  |  |  |  |  |  |
| Ischemic heart disease | 3,948 | 1 | 1.06 (0.96-1.18) | 1.01 (0.90-1.14) | 1.09 (0.96-1.23) | 0.311 |
| Ischemic stroke | 1,196 | 1 | 1.04 (0.86-1.26) | 0.99 (0.79-1.24) | 1.00 (0.79-1.26) | 0.914 |
| Haemorrhagic stroke | 3,542 | 1 | 1.09 (0.97-1.23) | 1.33 (1.17-1.52) | 1.16 (1.00-1.33) | 0.012 |
| Other CVDs | 2,238 | 1 | 1.11 (0.97-1.26) | 1.08 (0.93-1.27) | 0.99 (0.83-1.18) | 0.966 |
| Stomach cancer | 1,367 | 1 | 0.99 (0.83-1.19) | 1.07 (0.88-1.29) | 1.06 (0.87-1.28) | 0.517 |
| Oesophageal cancer | 1,017 | 1 | 0.96 (0.81-1.15) | 0.95 (0.75-1.22) | 1.42 (1.13-1.79) | 0.007 |
| Colorectal cancer | 759 | 1 | 1.24 (0.97-1.58) | 1.17 (0.90-1.51) | 0.99 (0.76-1.30) | 0.724 |
| Digestive tract cancer | 3,143 | 1 | 1.03 (0.92-1.15) | 1.05 (0.93-1.20) | 1.13 (0.99-1.28) | 0.074 |
| Non-digestive tract cancer | 7,249 | 1 | 0.96 (0.89-1.04) | 1.03 (0.95-1.11) | 0.97 (0.89-1.06) | 0.843 |
| Lung cancer | 2,626 | 1 | 0.96 (0.84-1.09) | 1.01 (0.88-1.15) | 0.97 (0.84-1.11) | 0.833 |
| Liver cancer | 1,566 | 1 | 0.97 (0.83-1.13) | 1.06 (0.89-1.26) | 0.89 (0.74-1.07) | 0.384 |
| All other non-digestive cancers | 3,057 | 1 | 0.96 (0.86-1.08) | 1.02 (0.90-1.15) | 1.01 (0.89-1.16) | 0.654 |
| COPD | 1,352 | 1 | 1.06 (0.86-1.29) | 0.95 (0.76-1.18) | 1.01 (0.80-1.27) | 0.831 |
| All respiratory diseases | 1,843 | 1 | 1.20 (1.01-1.42) | 1.04 (0.87-1.25) | 1.12 (0.92-1.35) | 0.607 |
| Other major chronic diseases | 1,986 | 1 | 0.98 (0.85-1.14) | 1.00 (0.85-1.17) | 0.87 (0.73-1.03) | 0.159 |
| Transport accidents | 1,001 | 1 | 0.98 (0.80-1.21) | 0.97 (0.77-1.23) | 0.84 (0.65-1.08) | 0.182 |
| All other causes | 2,479 | 1 | 1.03 (0.91-1.18) | 1.01 (0.88-1.17) | 0.96 (0.81-1.13) | 0.602 |

BMI, body mass index; CI, confidence interval; COPD, chronic obstructive pulmonary disease; CVD, cardiovascular disease; HR, hazard ratio.

^a^The multivariate-adjusted model was stratified by age-at-risk (5-year intervals), sex and study area (10 regions), adjusted for marital status (never married, married, divorced/separated, or widowed), body mass index (in kg/m^2^; continuous), household income (in yuan/year; <10,000, 10,000-19,999, 20,000-34,999, or ≥35,000), survey season, highest education level (no formal school, primary school, middle or high school, or college and above), physical activity (MET-h/wk), smoking (non-smoker, occasional smoker, ever-smoker, or current smoker), alcohol drinking (non-drinker, occasional drinker, ever-drinker, or current drinker), family history of cancer (yes or no), family history of CVD (yes or no), intake of red meat, poultry, eggs, fish, soybeans, fruit, dairy (never/rarely, monthly, 1-3 days/week, regularly) and fresh vegetables (daily or less than daily) at baseline.

^b^Dose of cigarettes for current smokers [<20, 20-24, and ≥25 cigarettes (or equivalent) per day] and dose of alcohol for current drinkers (<140, 140 to <420, and ≥420 g of alcohol per week) were controlled.
